# CPR: cardiac phosphatase in resuscitation

**DOI:** 10.1172/JCI169217

**Published:** 2023-05-01

**Authors:** Arjun Deb

**Affiliations:** 1 Division of Cardiology, Department of Medicine, David Geffen School of Medicine,; 2 Cardiovascular Theme, David Geffen School of Medicine,; 3 Department of Molecular, Cell and Developmental Biology,; 4 Eli and Edythe Broad Center of Regenerative Medicine and Stem Cell Research,; 5 Molecular Biology Institute, and; 6 California Nanosystems Institute, UCLA, Los Angeles, California, USA.

## Abstract

Out-of-hospital cardiac arrest is associated with a dismal mortality rate and low long-term survival. A large pharmacological knowledge gap exists in identifying drugs that preserve neurological function and increase long-term survival after cardiac arrest. In this issue of the *JCI*, Li, Zhu, and colleagues report on their engineering of a 20–amino acid cell-permeable peptide (TAT-PHLPP9c) that antagonized the phosphatase PHLPP1 and prevented PHLPP1-mediated dephosphorylation and AKT inactivation. TAT-PHLPP9c administration maintained activated AKT after arrest and led to AKT-mediated beneficial effects on the heart, brain, and metabolism, resulting in increased cardiac output and cerebral blood flow and rescue of ATP levels in affected tissues. TAT-PHLPP9c improved neurological outcomes and increased survival after cardiac arrest in murine and porcine models of cardiac arrest. These findings provide proof of concept that pharmacological targeting of PHLPP1 may be a promising approach to augmenting long-term survival after cardiac arrest.

## Sudden cardiac arrest

Sudden death or sudden cardiac death (SCD) has been defined as the occurrence of death within one hour of onset of symptoms in individuals with no prior conditions that would limit their life span and is secondary to sudden cessation of cardiac activity (sudden cardiac arrest [SCA]) with hemodynamic collapse (1). Despite advances in cardiovascular medicine, the annual incidence of SCD has largely remained unchanged and conservative estimates point to 230,000 to 350,000 deaths per year in the United States over the last 20 years (2, 3). More than 50% of all cases of sudden death occur in the community, as the first clinical event and out-of-hospital cardiac arrests have a dismal prognosis, with an estimated 10% overall survival rate (4). SCA occurring at home is associated with an even lower survival rate at 6%. Timely cardiopulmonary resuscitation (CPR), defibrillation of a shockable rhythm, and targeted temperature management using active cooling are some of the strategies used to improve survival (5). However even with return of spontaneous circulation (ROSC), many patients die within hours secondary to cardiovascular collapse, irreversible brain injury, and a systemic metabolic insult to many organs (6). There is thus a desperate need for developing pharmacological approaches for improving long-term survival after SCA.

## Targeting the AKT phosphatase PHLPP1

In this issue of the *JCI*, Li and Zhu report on their engineering of a peptide (TAT-PHLPP9c) that inhibited the phosphatase PHLPP1 and increased survival in murine and rodent models of cardiac arrest (7). The rationale for developing this therapeutic approach stems from earlier publications made by the authors and others demonstrating that organ protection from cooling initiated after SCA is mediated by the molecule AKT (7–9). In those studies, inhibition of AKT by using a pharmacological inhibitor or genetic loss of function resulted in loss of protection from cooling (10). Activation of AKT is regulated by phosphorylation events, and given the role of AKT in mediating organ protection after SCA, the authors pursued developing drugs that prevented deactivation of AKT after SCA, thereby maintaining activated AKT (7). The PH domain leucine rich protein phosphatase 1 (PHLPP1) is a member of the serine/threonine phosphatase family and known to dephosphorylate and inactivate AKT (11), and members of the PHLPP family have been considered as therapeutic targets for modulating AKT-dependent pathological phenotypes (12). Cardiac muscle cells deficient in PHLPP1 are resistant to doxorubicin or hydrogen peroxide–induced injury, and animals deficient in PHLPP1 exhibit increased AKT activation and cerebral protection following ischemia/reperfusion injury (11, 13). These observations formed the scientific premise for targeting the AKT phosphatase PHLPP1 to activate AKT and augment physiological function and recovery after cardiac arrest.

Li, Zhu, and authors engineered a 20–amino acid cell-permeable peptide comprising nine C-terminal amino acid residues of PHLPP1 and 11 amino acids of the cell membrane transduction domain of the TAT protein (7). The authors first showed that TAT-PHLPP9c peptide, when added to cardiac muscle cells, increased the amount of AKT that was phosphorylated at a specific site (Ser 473), a site that PHLPP1 targets for dephosphorylation. Notably, the peptide did not affect phosphorylation of AKT at other residues where PHLPP1 does not exert phosphatase activity, demonstrating the peptide to be a specific inhibitor of PHLPP1 in regulating AKT activation. The authors next studied the kinetics of the protein by labeling it with a GFP tag and showed that the protein could be detected in the heart and brain within five minutes of injection and was observed up to 60 minutes after delivery. As phosphorylation and dephosphorylation events occur rapidly, the distribution kinetics of the peptide thus enabled rapid activation of AKT in major organs (7). What remains unclear, however, is whether the peptide has greater affinity for the heart and brain than other organs or whether it is distributed in a nonselective manner to all organs after delivery.

Li, Zhu, and authors next tested the drug in a murine model of cardiac arrest and injected the peptide intravenously during CPR. In the control group, 36% of the animals had ROSC, but most of the animals died within 10 minutes of ROSC. In contrast, in animals that received the peptide, 82% had return of ROSC and survived substantially longer than the control group. Cerebral blood flow measured by magnetic resonance imaging was greater in the peptide-treated animals than in the saline-treated controls. Echocardiography also demonstrated superior contractile cardiac function with substantially increased ejection fraction or fractional shortening. Consistent with these findings, peptide-treated animals showed increased AKT phosphorylation, at the Ser473 residue, in both heart and brain within 15 minutes of ROSC. The animals also showed phosphorylation of GSK3β, which is downstream of and phosphorylated by activated AKT, suggesting that activated AKT initiates a beneficial signal transduction cascade (7). The mechanism of increased cerebral blood flow is not clear, and whether the peptide exerts an independent effect on cerebral vasculature or whether the effects are secondary to better cardiac function cannot be distinguished in the study (7). Notwithstanding, increased cerebral flow and increased cardiac contractile function after ROSC are important physiologic predictors of recovery, and the peptide delivers in this regard. Anaerobic metabolism occurs during tissue hypoxia, and increased blood flow is expected to decrease tissue hypoxia and promote a switch back to aerobic metabolism. In regard to increased blood flow and decreased hypoxia, Li, Zhu, and authors provide corroborative evidence of the salutary effects of the peptide, with the brain and heart tissues demonstrating increased ATP content and activation of pyruvate dehydrogenase (PDH), a key enzyme that regulates carbon entry into the TCA cycle for oxidative phosphorylation. However, the evidence presented falls short in determining whether changes in PDH phosphorylation and activity reflect a direct downstream effect of increased AKT activation or simply occur secondary to increased tissue-blood flow and attenuation of tissue hypoxia (7).

Encouraged by these observations, Li, Zhu, and colleagues performed two seminal experiments to definitively determine the role of TAT-PHLPP9c in survival after cardiac arrest. They performed a randomized double-blinded study in which they injected the peptide or saline during CPR into two groups of mice subjected to cardiac arrest. At five days after cardiac arrest, the number of surviving animals was higher in the peptide-injected group and this group showed greater mean arterial blood pressure (MAP) as well as superior neurological function. Next, the authors used a porcine model of ventricular fibrillation and demonstrated that the peptide-injected animals had superior survival. Only one of eight animals injected with saline had ROSC, while 80% of the animals that received the peptide achieved ROSC. Neurological recovery was also superior in the peptide-injected group, and MAP was closer to prearrest values after ROSC in the TAT-PHLPP9c–injected animals. Finally, the authors measured two metabolites, taurine and glutamate, that can be released from stressed or injured tissues and demonstrated decreased circulating levels of plasma taurine and glutamate after ROSC in mice that received the peptide. The authors suggest that inhibition of release of taurine and glutamate could help in more rapid replenishment of cellular stores, thereby promoting a more rapid functional recovery of the heart and brain (7).

## Conclusions

**Figure 1:**
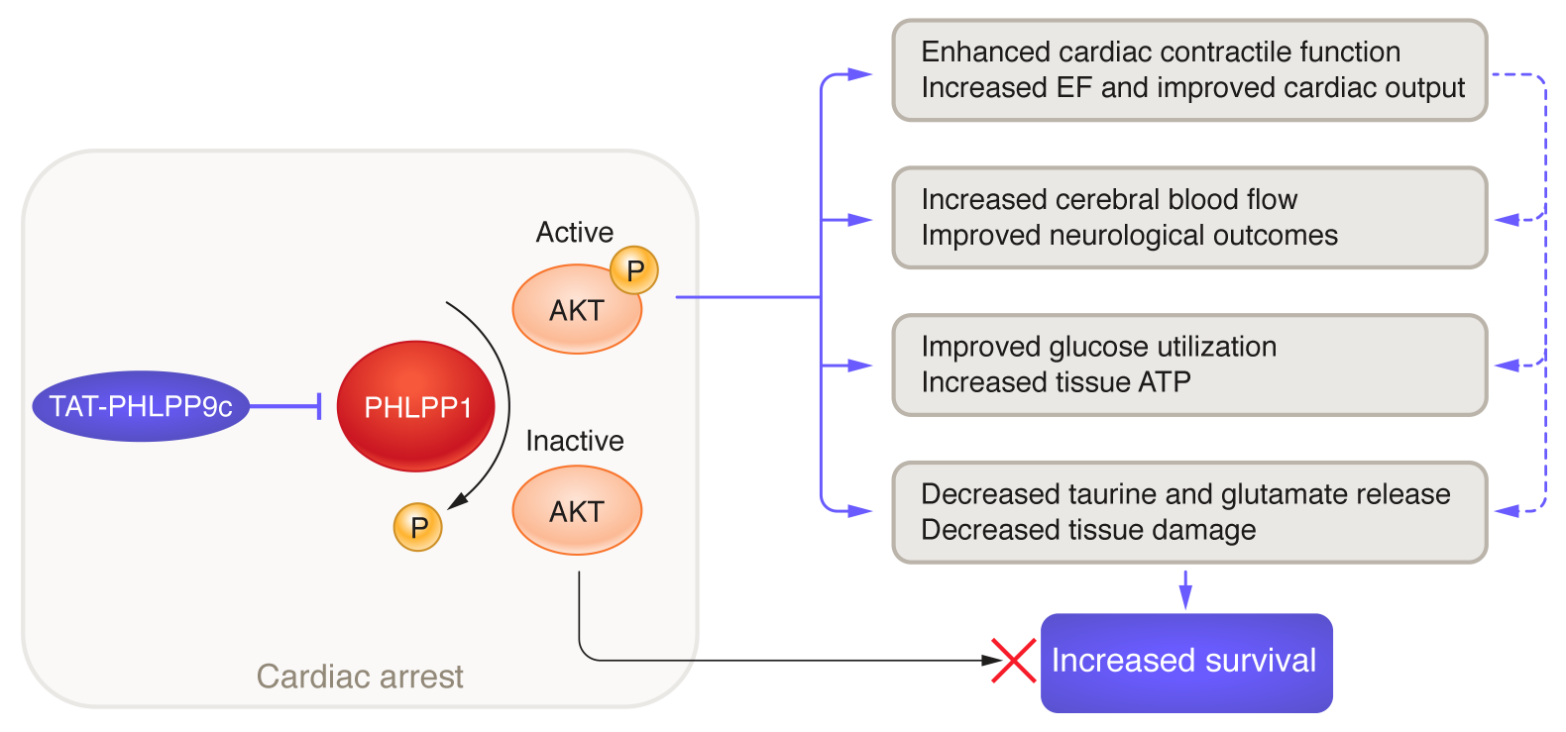
Inhibition of PHLPP1 using the 20–amino acid peptide TAT-PHLPP9c augments brain, heart, and metabolic function after cardiac arrest in preclinical models. Following cardiac arrest, PHLPP1 dephosphorylates and inactivates AKT at Ser473. TAT-PHLPP9c peptide inhibits PHLPP1-mediated dephosphorylation of AKT, which leads to maintenance of Ser473 phosphorylation and activation of AKT. Activated AKT mediates beneficial effects on the heart, brain, metabolism, and damaged tissues. Secondary beneficial effects (dashed arrows) are mediated by increased cardiac output, increased blood flow in organs, and attenuation of tissue hypoxia. Collectively, these effects of maintaining activated AKT lead to increased survival after cardiac arrest. EF, ejection fraction.

Li and Zhu’s paper (7) is an important study that addresses a large knowledge gap in identifying drugs that preserve or prevent organ injury after cardiac arrest (Figure 1). Although some specifics are not provided, such as detailed peptide kinetics, distribution of the peptide, and signal transduction cascades downstream of AKT, these deficits, in my opinion, do not constitute major drawbacks of the study, but rather represent questions the authors will need to address in future studies. Indeed, many other questions arise: What are mechanisms of increased cerebral blood flow and improved cardiac function? Are beneficial effects on cellular metabolism autonomous of blood flow? And what are the direct effects of the peptide on hypoxic organ injury during arrest? It is also unclear how this drug will synergize with cooling techniques that are currently implemented after cardiac arrest, where the goal is to lower organ metabolic rates to preserve organ function and minimize injury (14). A peptide that increases cellular metabolism and promotes rapid resumption of oxidative phosphorylation, as the authors suggest, may be at odds with strategies that aim to lower metabolic rates. Such issues would need well-designed, preclinical models and human trials to determine the temporal window of optimal therapeutic benefit for TAT-PHLPP9c.

The pharmacology of drugs acutely administered to treat out-of-hospital cardiac arrest is complex. For instance, epinephrine, a commonly administered drug, was shown to improve 30-day survival, but was associated with worse neurological outcomes among survivors (15). Antiarrhythmic drugs have also not been convincingly demonstrated to increase long-term survival after out-of-hospital cardiac arrest (16). Li and Zhu’s work (7) brings us hope that AKT activation using a pharmacological inhibitor of cardiac phosphatase will improve long-term survival and neurological outcomes after CPR (Figure 1).

## Acknowledgement

This Commentary was supported by grants from the NIH (HL149658, HL152176, HL149687, AR075867, and DK132735).

## Version 1 05/01/2023

Electronic publication
